# Supplementary material for: Which Actigraphy Dimensions Predict Longitudinal Outcomes in Bipolar Disorders?
Source: J Clin Med. 2022 Apr 14;11(8):2204. doi: 10.3390/jcm11082204 (PMC9027161; doi:10.3390/jcm11082204)
Supplement: Supplementary file 1 [file jcm-11-02204-s001.zip › jcm-1650279-supplementary.pdf]

Supplementary Materials

Table S1.PCA (Principal Component Analysis) of sleep quality actigraphy estimates

Variables *	SQ1	SQ2	SQ3
FI	0.92		
WASO	0.88		0.50
WASO SD	0.85		
FI SD	0.81		
SE	-0.81		
TST SD	0.61		
SE SD	0.56		-0.51
SOL SD		-0.92	
SOL		-0.91	
TST			0.85

*: standardized values, SQ: Sleep Quality factors
TST: total sleep time, SOL: sleep onset latency, WASO: time spent awake after sleep onset, SE: sleep efficiency, FI: Fragmentation Index, SD: Standard Deviation
PCA was performed with an Oblimin rotation.
KMO=0.60; Bartlett's Test of Sphericity: Chi-square: 639, df28, $p=10^{-87}$

Table S2. PCA (Principal Component Analysis of circadian rhythms actigraphy estimates

Variables *	CR1	CR2	CR3
M10	0.98		
Amplitude	0.95		
IV	-0.78		
IS	0.62		
L5onset		0.91	
M10onset		0.91	
L5			-1.01
Relative amplitude			0.89

*: standardized values, CR: Circadian Rhythms factors
IS: inter-day stability, IV: intra-day variability
PCA was performed with an Oblimin rotation.
KMO=0.60; Bartlett's Test of Sphericity: Chi-square: 545, df45, $p=10^{-17}$

Table S3. Duration of follow-up and attrition rates

Duration of follow-up	Number of participants	Attrition rate (%)
At least 12 months	8	11.6 %
At least 24 months	8	23.2 %
At least 36 months	13	42 %
At least 48 months	15	63.8 %
At least 60 months	25	100 %

Table S4. Multivariable survival analysis (Cox regression model) using actigraphy variables contained in Circadian Rhythms Factor 1

Variables	Beta	SE	Wald	df	p	HR	Lower 95%CI	Upper 95%CI
Age	0.023	0.015	2.462	1	0.12	1.02	0.99	1.05
Type BD	-0.421	0.385	1.195	1	0.27	0.66	0.31	1.39
**MS Polytherapy**	0.973	0.336	8.406	1	**0.004**	2.65	1.37	5.11
Density Mood Episodes*	1.115	0.659	2.864	1	0.09	3.05	0.84	11.08
**BMI**	-0.136	0.053	6.476	1	**0.011**	0.87	0.79	0.97
**IV**	1.537	0.615	6.254	1	**0.012**	4.65	1.39	15.51

*: log-transformed

SE: Standard Error, HR: Hazard Ratio, CI: Confidence Interval, BD: Bipolar Disorder, BMI: Body Mass Index, IV: intra-day variability, MS: Mood Stabilizers

Table S5. Values and 95CI of the AUC of early versus late recurrence when classified using key clinical and circadian variables alone and in combination.

Variables	AUC	SE	p	Lower 95CI	Upper 95CI
Clinical variables *	0.64	0.07	0.045	0.50	0.78
IV	0.75	0.07	0.0002	0.62	0.88
Clinical variables * + IV	0.82	0.05	10^{-9}	0.72	0.92

* Clinical variables included age, BD type, MS polytherapy, BMI, Density of mood episodes.

AUC: Area Under the Curve, SE: Standard Error, CI: Confidence interval
